# Evaluation of a student-run smoking cessation clinic for a medically underserved population

**DOI:** 10.1186/1756-0500-4-55

**Published:** 2011-03-08

**Authors:** Lindsay E Lough, Jon O Ebbert, Thomas G McLeod

**Affiliations:** 1 Mayo Clinic, 200 1st Street Southwest, Rochester, MN 55905, USA

## Abstract

**Background:**

Smoking is common among medically underserved populations. Accessible resources to encourage and support smoking cessation among these patients are limited. Volunteer medical student-run free smoking cessation clinics may provide an effective option to help these individuals achieve smoking abstinence. In order to demonstrate the feasibility and cost-effectiveness of a student-run clinic, we analyzed a case series of patients receiving care in a medical student-run Smoking Cessation Clinic (SCC) at the Rochester, Minnesota Salvation Army Good Samaritan Health Clinic (GSHC).

**Findings:**

Between January 2005 and March 2009, 282 cigarette smokers seeking care at the SCC were analyzed. Student providers at the SCC conducted 1652 weekly individual counseling sessions averaging 18 minutes per encounter. Patients were offered a choice of pharmacotherapies including nicotine replacement therapy (NRT), bupropion, and varenicline for up to 12 weeks. Smoking abstinence was confirmed with exhaled carbon monoxide (CO). Thirty-two patients completed the entire 12-week program (11.3%). At last contact, 94 patients (33.3%) abstained from smoking for ≥ 7 days and 39 patients (13.8%) were continuously abstinent for ≥ 4 weeks. The 7-day point prevalence abstinence rates at last contact were 58.6% for varenicline, 41.2% for bupropion, 33.9% for NRT, and 23.5% for bupropion and NRT. Analyzing missing patients as smoking, the 7-day point prevalence abstinence rates were 7.1%, 8.9%, and 8.2%, at 1 month, 2 months, and 3 months after program enrollment, respectively. No serious adverse drug reactions were recorded.

**Conclusions:**

Our medical student-run smoking cessation clinic provided an effective and safe experience for medically underserved patients who might not otherwise have access to conventional smoking cessation programs because of high cost, lack of insurance, or other disparities. Similar medical student initiatives focusing on healthy lifestyles may be feasible and beneficial for individuals with limited access to healthcare resources.

## Background

Cigarette smoking is the leading cause of preventable death and disability in the United States. In 2008, 20.8% of the U.S adult population smoked with much higher rates among medically underserved and uninsured populations [1-3]. Among uninsured individuals in an urban public hospital setting, the smoking prevalence is 65% [4].

Numerous treatments for tobacco dependence exist [5]. Behavioral and pharmacologic approaches have demonstrated efficacy for increasing tobacco abstinence rates. Providing treatment for medically underserved populations, including financially challenged individuals and those without medical insurance, poses special challenges. For such individuals, access to conventional healthcare resources is limited and the cost of pharmaceuticals is often prohibitive [6-9]. High rates of psychiatric co-morbidity, substance use, and medical non-adherence further complicate the delivery of tobacco treatment services to underserved populations [10]. Prior investigations have demonstrated lower smoking cessation success rates for such populations [11-13].

Medical student-run clinics have been shown to benefit both the education of the students and the community serviced by the clinic [14-16]. To increase accessibility to tobacco use treatment for medically underserved populations, Mayo Medical School established a free, walk-in Smoking Cessation Clinic (SCC) in partnership with the Rochester, Minnesota, Salvation Army. The clinic is staffed and managed by volunteer medical students working on-site at the Salvation Army's Good Samaritan Health Clinic (GSHC).

In 2001, we reported our preliminary experience at the clinic and highlighted the feasibility of this care model [17]. In the current report, we describe the patient demographics and smoking cessation outcomes from a large cohort of patients seen at the SCC. We also present data on the cost of running a student-led smoking cessation clinic for the medically underserved.

## Methods

### Study Setting

The Smoking Cessation Clinic (SCC) is in Rochester, Minnesota, with a catchment area of approximately 140,000 people of whom 89% are white, 3.7% are African American, 5% are Asian, and 0.3% are Hispanic [18]. The current unemployment rate for the community is 5.4% [19]. Approximately 8.7% of residents have no health insurance [20].

The SCC is located in the Rochester Salvation Army's Good Samaritan Health Clinic (GSHC). The GSHC was established in 1995 by the Salvation Army in partnership with Mayo Clinic physicians to voluntarily provide selected free healthcare services to medically underserved individuals in the community. Clinic services include acute care, selected chronic disease management (e.g., diabetes), dental services, ophthalmologic screening, and psychiatry/psychology assessment and support.

The study took place between January 2005 and March 2009. SCC sessions are conducted after hours one night a week coinciding with acute care clinical activities of the GSHC. Licensed medical providers and on-site pharmacy services of the GSHC are available to SCC student counselors as necessary.

### Patient Enrollment

The SCC is advertised to the community with print and radio advertising. Patients seen by providers in other GSHC areas (e.g., GSHC Dental Clinic) are referred to the SCC as appropriate. Although both the SCC and GSHC are oriented toward providing services to uninsured individuals of lower socioeconomic status, all patients requesting smoking intervention services can receive services regardless of their financial status, current health conditions, or stated motivation to complete the program.

### Individualized Therapy

Medical student volunteers are trained using an apprenticeship model with minimal physician intervention. Experienced second year medical student counselors train first year volunteer medical student counselors by modeling SCC interviews. The second year counselors then observe first year counselors interviewing patients and provide feedback on provider-patient interactions. Activities at the SCC are regularly reviewed by a physician staff specialist of the Mayo Clinic Nicotine Dependence Center.

At the first visit, each patient completes an 8-page intake questionnaire addressing tobacco use history, demographic information, and smoking-related or psychiatric health conditions. Patients then meet individually with a student counselor for approximately 45 minutes so that the patient and counselor may discuss program structure, cigarette use history, and other elements of the intake assessment.

Depression screening is completed using the Center for Epidemiological Studies - Depressed Mood Scale (CES-D). This instrument has been found to have good internal consistency and concurrent validity. It has been shown to be appropriate for use in populations with a broad range of demographic characteristics [21]. The intake assessment also includes a detailed checklist to screen for contraindications to pharmacotherapy. The checklist of contraindications is verbally read to each patient to ensure patient understanding of each question.

Patients are given the option of individual counseling only or counseling in conjunction with varenicline, bupropion, and/or nicotine replacement therapy (NRT). Patients who request and qualify for NRT receive their choice of a 7-day supply of patches, gum, lozenges, inhalers, or nasal spray according to their preferences on the first intake visit. Medical prescriptions are signed by an on-site licensed volunteer GSHC provider. Intake assessment and management plans for all enrolled subjects are reviewed and approved by a physician specialist of the Mayo Clinic Nicotine Dependence Center. Financial support is provided by Mayo Clinic and medications are supplied by pharmaceutical companies.

Patients are invited to return to the SCC for weekly follow-up counseling sessions for the next 12 weeks of the program. During these 15-30 minute sessions, counselors discuss management of cravings and withdrawal symptoms as well as encourage patients to make lifestyle modifications in order to address the behavioral aspects of smoking addiction. Appropriate medication use and potential adverse effects are reviewed with the patient and pharmacotherapy is modified as necessary. Finally, counselors partner with patients to establish weekly goals for continued progress towards complete smoking cessation. At the end of each session, the counselors complete a weekly follow-up form documenting the patient's smoking cessation progress, motivation, and medication side effects. An additional 7-day supply of the appropriate medication is dispensed if requested by the patient, and the patient is encouraged to return the following week. Patients were requested to return for weekly sessions for 12 weeks. At the end of 12 weeks, patients who complete the program are tapered off their medications as appropriate.

### Data Collection

Each subject completes general research authorization during their first visit with the understanding that refusal to authorize research participation does not affect their current or future care at either the SCC or the GSHC. Minors (i.e., age < 18 years) and those who refused participation are excluded from the current analysis. Participant charts were abstracted for sociodemographic data, smoking history, and program progress. Self-reported abstinence was biochemically confirmed by counselors with exhaled carbon monoxide (CO).

### Statistical Methods

Baseline patient demographic information, health history, and tobacco use patterns were recorded at initial intake. Clinic utilization data, patient tobacco use, and dispensed medication were recorded from weekly follow-up forms. Descriptive statistics were used to compile means, standard deviations (SDs), frequencies, and percentages for all enrolled subjects. The number and percentage of patients given each medication were calculated as the number/percentage of patients who received that medication during the course of their therapy even if they later switched to a different medication.

Smoking abstinence was defined as 7-day point prevalence abstinence (i.e., no smoking, not even a puff in the last 7 days) and continuous abstinence (i.e., no smoking for ≥ 4 weeks) prior to their last contact with SCC. Abstinence rates were compiled for the entire study population and time period. Subgroup analyses were conducted in order to test for the effect of the release of varenicline on smoking abstinence rates, to examine temporal trends in patient abstinence rates on a yearly basis, and to examine the effects of each treatment type on abstinence rates. Specifically, subgroup analyses included abstinence rates for: 1) the study population before and after February 2006, the date on which varenicline became available for use at the SCC; 2) each individual year from 2005-2008; and 3) each treatment type (varenicline, bupropion alone, NRT alone, or a combination of bupropion and NRT). Abstinence rates for each treatment type were calculated as the percentage of patients abstinent at the time of last contact out of the total number of patients who took that treatment type. Abstinence rates were calculated for treatment type such that an individual subject could be counted in two different treatment groups if they received more than one type of therapy over the study timeframe. Abstinence rates were also calculated for the total patient population using an intention-to-treat analysis for 1-month, 2-month and 3-month time points since program enrollment in which all patients who did not return for their appointments were counted as smoking.

To test for demographic characteristic differences among smokers who chose different treatment plans, we conducted ANOVA analysis for continuous baseline variables (age and cigarettes smoked per day at the beginning of treatment) as well as a chi-square test for categorical variables (sex and motivation level). Although there were 5 treatment groups, there were very few people who received bupropion alone (n = 1). We restricted this analysis to patients who were only on bupropion+NRT and never switched to any other therapy (n = 5). Therefore, when comparing the other baseline variables across treatment groups, we eliminated these groups and compared the variables across the other 3 treatment groups (varenicline, NRT, and combination therapy). Combination therapy was defined as a patient who began treatment with one medication but later changed to a different medication during the course of treatment.

Yearly budgets for 2005, 2006, 2007, and 2008 were calculated based on the average cost of medication distributed to patients for that year. The average cost-per-quit was calculated by averaging the yearly cost of running the clinic by the number of patients with 7-day point prevalence smoking abstinence at their 3-month time point since program enrollment adjudicating all patients not present as smoking.

## Results

### Demographic Data

Of 282 cigarette smokers who sought care at the SCC between January 2005 and March 2009, charts could be abstracted for baseline demographics and tobacco use history data for 257 patients (Table 1). Over a six month period in 2008-2009, the SCC saw an average of six patients per week. Extrapolating this data across January 2005 to March 2009, approximately 1320 patients were treated of which 21.4% (282) participated in the study. The average age of smokers was 42 years (SD ± 13.1) and 45% were female. Patients smoked an average of 27 cigarettes per day (SD ± 14.5) and had been smoking for an average of 24 years (SD ± 12.1 years).

**Table 1:** Demographics of Patients Receiving Care at the Salvation Army Smoking Cessation Clinic*

	N (%)		N (%)
Men	140 (54.5)	Other Smoker in Household	67 (26.1)

Women	113 (44)		

Education		Tobacco Products	

<8th grade	3 (11.7)	Cigarettes	251 (97.7)

>8th grade	2 (0.1)	Pipe	1 (0.1)

Some High School	18 (7)	Cigar	6 (2.3)

High School Degree	101 (39.3)	Snuff	2 (0.1)

Technical Degree	75 (29.2)	Chew	6 (2.3)

College Degree	41 (15.9)	Major Stressors	

Graduate Degree	8 (3.2)	Death	30 (11.7)

Other	3 (1.2)	Loss of relationship	25 (9.2)

Employment		Divorce/Separation	17 (6.6)

Health Care	15 (5.8)	Health	57 (22.2)

Professional	11 (4.3)	Job	28 (10.9)

White Collar	29 (11.3)	Move	19 (7.4)

Blue Collar	67 (26.1)	Legal	17 (6.6)

Homemakers	4 (1.5)	Other	68 (26.5)

Unemployed	55 (21.4)	None	71 (27.7)

Retired	14 (5.4)	Depression	

Student	13 (5.1)	>2 wks/yr with sad feelings	108 (42)

Other	49 (19.1)	>2 yrs with sad feelings	90 (35.1)

Ethnicity		Sad most of this year	77 (30)

Native American	9 (3.5)	Treated for depression	117 (45.5)

Asian	0	Motivation	

African American	9 (3.5)	None	0

Hispanic	4 (1.6)	Some	45 (17.5)

White	227 (88.0)	Very	205 (79.8)

Other	1 (0.1)	Previous Cessation Techniques	

Medical History/Complaints		Abrupt cessation	152 (59.1)

Angina	13 (5.1)	Self-help	55 (21.4)

Asthma	59 (23)	Counseling	30 (11.7)

Cancer	16 (6.2)	Reduction	78 (30.3)

Chest Pain	57 (22.2)	Hypnosis	29 (11.3)

Bronchitis	49 (19.1)	Acupuncture	9 (3.5)

Diabetes	17 (6.6)	Formal program	22 (8.6)

Emphysema	16 (6.2)	Inpatient program	4 (1.6)

Cough/Chest Colds	84 (32.7)	Nicotine gum	93 (36.2)

MI	11 (4.3)	Nicotine patches	153 (59.5)

Morning Cough	133 (52)	Bupropion	80 (31.1)

Other	3 (1.2)	Group therapy	9 (3.5)

		Nicotine nasal spray	6 (2.3)

		Nicotine inhaler	54 (21)

* Data is from January 2005 - March 2009 (N = 257)

^ ± ^All patients who used another form of tobacco other than cigarettes also concurrently used cigarettes.

A majority of patients (74.6%; n = 192) reported at least one smoking related medical condition and were coping with at least one major stressor in their lives (72.4%; n = 186). Nearly one-half of patients received formal treatment for major depression and a third of patients reported "feeling sad" for most of that year. Almost all patients reported consuming alcohol at one point in their life (96.1%; n = 247) and over one-quarter of patients felt that they needed to decrease their alcohol intake (26.1%; n = 67). Over one-quarter of patients had also entered into a formal treatment program for drug abuse (28.4%; n = 73), although the majority of patients stated that they had been drug free for greater than one year at the time of initial treatment (93.4%; n = 240).

In general, the patient population self-reported that they were highly motivated to quit smoking (79.8%; n = 205). Most patients had a desire to quit for >1 year (56%; n = 144) and most patients (69.3%; n = 164) had made at least 2 to 5 quit attempts in the past.

### Treatment and Follow-Up

Patients could receive more than one type of medication therapy. Among the patients receiving pharmacotherapy (N = 282), 60.0% (n = 169) of patients received varenicline, 40.0% (n = 112) received NRT alone, 12.1% (n = 34) received combination therapy with bupropion and NRT, and 6.0% (n = 17) received bupropion alone. No significant differences were observed between treatment groups for any of the characteristics evaluated (sex p = 0.30, motivation p = 0.95, age p = 0.13, cigarettes per day p = 0.22).

### Smoking Abstinence

Of 282 patients, 94 (33.3%) successfully quit smoking for ≥ 7 days prior to last contact and 39 (13.8%) were continuously abstinent for ≥ 4 weeks at time of last contact. Although only 54.3% (n = 153) of patients attended the fourth treatment session, 29.8% (n = 84) of the patients successfully abstaining from smoking had quit by one month after initial consultation. Almost one-third (28.7%; n = 81) of patients did not return after their second visit.

Before the clinic began offering varenicline in February 2006, 83 patients received treatment at SCC of whom 18.0% (n = 15) successfully abstained from smoking ≥ 7 days at last contact. After the clinic began dispensing varenicline, 199 patients were treated at SCC before March 2009 of whom 39.7% (n = 79) achieved 7-day point prevalence smoking abstinence at last contact.

The overall clinic 7-day point prevalence smoking abstinence rates at last contact were as follows: 19.2% in 2005 (14/73 patients); 22.2% in 2006 (16/72 patients); 42.9% in 2007 (36/84 patients); and 46.9% in 2008 (23/49 patients).). The 7-day point prevalence abstinence rates for the different medications were as follows: 58.6% (99/169 patients) for varenicline; 41.1% (7/17 patients) for bupropion alone; 33.9% (38/112 patients) for NRT alone; and 23.5% (8/34 patients) for bupropion and NRT.

Abstinence rates were also determined at monthly time points for program participation. Of 113 patients seen at their 1-month follow-up visit, 20% (n = 23) achieved 7-day point prevalence smoking abstinence. Of 56 patients presenting for their 2-month follow-up visit, 25% (n = 14) were abstinent. Of the 40 patients presenting for their 3-month follow-up, 23% (n = 9) were abstinent. Of the entire patient population with subjects not returning adjudicated as smoking, the 7-day point prevalence abstinence rates for 1-, 2-, and 3-month follow up visits were 7.1% (n = 20), 8.9% (n = 25), and 8.2% (n = 23), respectively.

We also analyzed correlations between patient demographics and attendance at counseling sessions. While 25.7% (n = 44) of the 169 patients who returned for their third visit had received treatment for illicit drug use, 35.7% (n = 30) of the 84 patients not returning for their third visit had received treatment. Similarly, while 100% of those patients who returned for their third visit had been abstinent from illegal drugs for >1 year, only 80% (n = 67) of those not returning had been abstinent.

### Budget

The total budget spent on medication given to SCC patients during the study time period was $51,272. The yearly budgets for 2005, 2006, 2007, and 2008 were $8,615, $12,234, $20,396, and $10,027, respectively. The average cost per successfully abstinent patient at the 3-month time point was $1231, $1529, $2266, and $1671 for 2005, 2006, 2007, and 2008 respectively.

## Discussion

We observed that a student-run smoking cessation clinic is feasible and can facilitate smoking abstinence among medically underserved populations. One-third of the patients were abstinent from smoking at last contact. Although the drop-out rate was high, 54.4% of patients attended at least four treatment sessions.

Our smoking cessation clinic run by medical students achieved 7-day point prevalence and long-term abstinence rates comparable to those reported for other conventional treatment programs [22-24]. We believe that these results are notable given that they were achieved in a medically underserved population with demographic predictors of non-adherence such as high rates of psychiatric illness, substance abuse, and serious health conditions.

In an attempt to be consistent with the rest of the tobacco treatment literature, we have adjudicated subjects lost to follow-up as smoking. This is a conservative estimate and we recognize that this likely underestimates the true tobacco abstinence rate. However, this conservative estimate underscores the critical importance of increasing the focus on tobacco use among underserved populations who represent the demographic with the highest prevalence of use. Benowitz et al. noted that active smoking was particularly high among African American populations (77%), the uninsured (65%), self-reported alcohol drinkers (77%), and illicit drug users (90%) in an urban public hospital in San Francisco [4]. Public health efforts should continue to direct resources to the treatment of tobacco use in this group in order to decrease the tremendous death and disability caused by tobacco use and dependence.

Our results were accomplished within a reasonable budget. Our cost per patient who had abstained from smoking for 7 or more days at 3 months since initiation of treatment was between $1000-2000. This figure compares favorably to the $2000-3000 cost-per-quit figures previously published [25,26]. The cost-effectiveness of the clinic may be partially attributable to the provision of individualized counseling and the incorporation of the cost-effective medication varenicline at no charge to patients.

The feasibility of our clinic was initially reported in 2001 [17]. Results from the current analysis demonstrate marked improvement in abstinence rates with successive years of clinic operation. Incorporation of varenicline into our therapeutic armamentarium could certainly have contributed to this finding. Prior investigations have suggested that varenicline may be a more effective smoking cessation adjunct for some populations [27]. However, the addition of varenicline in February 2006, does not temporally correlate with the 20% increase in 7-day point prevalence abstinence rates at the time of last patient contact from only 22.2% in 2006 (16/72 patients) to 42.9% in 2007 (36/84 patients). It is possible that this delayed increase in abstinence rates may be related to delayed adoption of varenicline as a commonly prescribed pharmacotherapy. However, we suggest that the "apprenticeship" model used to orient new counselors may have contributed to the long-term success and improvement of the clinic over time. New counselors are oriented through a protocol that incorporates interview modeling, shadowing, and direct observation with pointed feedback on initial counselor-client interactions by a seasoned student provider. This method provides the opportunity for new counselors to learn from the experience of previous counselors, thereby continually improving the operation of the clinic. It also provides the experienced counselor with a teaching opportunity to pass on acquired knowledge. Reports of other student-staffed endeavors support the value of learning by this means [17,28,29].

We observed that the abstinence rates with the combination of NRT and bupropion were lower than with bupropion alone. In our clinic, counselors and subjects agree on treatment plans that are tailored for individual patients. We hypothesize that subjects selecting bupropion+NRT have relapsed with other drug regimens and are more tobacco-dependent resulting in lower observed abstinent rates on the combination.

Particular strengths of this study include the longevity of the clinic (14 years), the large number of enrolled subjects, a multi-faceted treatment plan (counseling and medication therapy), a reasonable budget, and biochemically-confirmed smoking abstinence.

Our study has several limitations. First, this is a retrospective cohort study. Since only patients who signed the consent form were included, data from patients with less motivation to both quit and participate in our research study may have increased our observed abstinence rates. Secondly, the design of the current trial does not allow us to draw any conclusions about the effect of a particular medication or treatment approach in this population of tobacco users. Patients were able to select the medications they used which may have improved adherence leading to improved efficacy. Our careful screening procedures prevented patients with a psychiatric history from receiving varenicline which may have elevated the observed abstinence rates among patients receiving varenicline. Third, abstinence rates were challenging to ascertain in this patient population because of the high drop out rate and skipped visits. As a result, we submit that the most informative efficacy rate for this study may be the reported 7-day point prevalence abstinence rates at the time of last patient contact with SCC. We selected the 7-day point prevalence abstinence rate to be consistent with the medical literature. However, this index captures only a one week window in the experience of a smoker attempting to become tobacco free and this measure may be an inadequate predictor of long-term abstinence. This estimate also includes a more heterogeneous population of smokers such as smokers who have quit for one week and smokers who have quit for one month unlike other measures such as continuous abstinence [30]. Finally, while we are not aware of any investigations demonstrating the efficacy of the mentor modeling teaching method in medical students, studies have shown that both peer mentoring and supervisor mentoring are successful in other populations [31,32]. Our cost analysis is limited by: 1) only the cost of medication actually distributed to patients was included and undistributed medications lost to expiration were not included; and 2) much of the medication used by patients at the SCC was donated by pharmaceutical companies.

We submit that the SCC has been successful in two primary ways: 1) the observed smoking abstinence rates among an underserved population are comparable to those observed in other studies; and 2) the clinic was a public service conducted by medical students reaching populations who would otherwise not have received these services allowing for new learning opportunities for medical students. Factors that may have contributed to the relative success of the clinic are: 1) the convenient time and location (i.e., after normal business hours, in a medical clinic located in a subsidized housing unit, near public transportation); 2) dynamic program that includes free weekly, individualized counseling sessions with dedicated and experienced counselors; and 3) free access to the spectrum of currently recommended smoking cessation medication. These strategies effectively eliminate some of the major barriers to smoking cessation treatment for medically underserved patient populations.

## Conclusions

Our study supports the feasibility and efficacy of a free, medical student led smoking cessation clinic with physician training involvement, a dedicated physician to oversee prescription management, and proper funding. It may serve as a model upon which future medical student-run free smoking cessation clinic endeavors could be based. Such endeavors have the potential to provide both an educational benefit to the student and a valued healthcare service to the community.

## List of Abbreviations

SCC: Smoking Cessation Clinic; NRT: nicotine replacement therapy; GSHC: Good Samaritan Health Clinic.

## Competing interests

JOE has received consulting fees in the last 12 months from GlaxoSmithKline (GSK). GSK had no involvement in any stage of this project. Medications for the clinic were provided by the Mayo Clinic. There are no other financial or non-financial competing interests.

## Authors' contributions

LEL conceived of the study, conducted the chart review and data analysis, and drafted the manuscript. JOE assisted with study design and provided critical input for manuscript writing. TGM reviewed and edited the manuscript, and oversees clinical activities at the GSHC (current Medical Director). All authors have read and approved this manuscript.
